
JOURNAL INFORMATION
==============================
NLM Title Abbreviation: Behav Neurol
Journal ID: Behav Neurol
NLM Journal ID: 8914585
Journal ID: BN

Behavioural Neurology

ISSN: 0953-4180
EISSN: 1875-8584
Publisher: Wiley

ARTICLE INFORMATION
==============================
PMCID: PMC5478844
PMID: 16410624
DOI: 10.1155/2005/492935
Article ID: 492935
Article version: 1
Subjects: Introduction

Acquired Disorders of Reading and Writing: Cross-Script Comparisons

Weekes Brendan Stuart
Department of Psychology School of Life Sciences University of Sussex Falmer Brighton BN1 9QG UK
*Brendan Stuart Weekes: bsw@biols.susx.ac.uk
Print publication date: 2005
Electronic publication date: 2006 Jan 9
Volume: 16
Issue: 2-3
First page: 51
Last page: 57
Received 2005 Dec 15; Accepted 2005 Dec 15
Copyright: Copyright © 2005 Hindawi Publishing Corporation and the authors.
Copyright year: 2005
License: This is an open access article distributed under the Creative Commons Attribution License, which permits unrestricted use, distribution, and reproduction in any medium, provided the original work is properly cited.
License URL: https://creativecommons.org/licenses/by/3.0/

Keywords: Models of reading, spelling, sound, regularity, consistency, bilingualism

==============================
Behavioural Neurology 16 (2005) 51–57
IOS Press

### Introduction

### Acquired disorders of reading and writing: Cross-script comparisons

Brendan Stuart Weekes
*Department of Psychology, School of Life Sciences, University of Sussex, Falmer, Brighton BN1 9QG, UK*
*Tel.: +44 1273 678058 (ex 8570); Fax: +44 1273 678611; E-mail: bsw@biols.susx.ac.uk*

Keywords: Models of reading, spelling, sound, regularity, consistency, bilingualism

Aphasiologists have accumulated a sophisticated understanding of the language impairments among English speakers. Research in the field of cognitive neuropsychology has improved our knowledge about how language becomes impaired in English speakers following brain damage by generating conceptual frameworks for understanding why language impairments occur. A large proportion of the research evidence that supports these models comes from studies of patients who have acquired problems in spoken and written naming (anomia), reading (dyslexia) and writing (dysgraphia). The ultimate aim of this research is to enhance the lives of patients with aphasia by improving diagnosis of language impairments and by development of innovative rehabilitation strategies.

One outstanding issue in the field is how to exploit our advances in knowledge to other languages. Cross-linguistic studies are rare but from what we do know there can be remarkably similar patterns of language impairment following damage to specific brain regions i.e., Broca's and Wernicke's areas that are known to be necessary for language comprehension and production [39,42,43]. However, the early work of pioneers such as Martin Albert, Liz Bates and Loraine Obler has taught us that communication disorders must be investigated in the context of the unique linguistic properties that characterize a particular language [1]. The challenge to researchers in the field of behavioural neurology is to document how these unique linguistic properties impact on specific communication abilities. Such reports may contribute to a cognitive neuropsychological framework used to interpret patterns of aphasia within languages. In addition to improving the lives of patients, this endeavor has the potential to lead to discoveries about how the brain adapts to different language environments. For example, we now know that distinct brain areas are implicated in dyslexia in different orthographies such as Chinese and English [54].

Much of the progress in our understanding of communication disorders in English comes from cognitive neuropsychological studies of patients with selective disorders of reading, spelling and writing. For example, acquired surface dyslexia in English refers to a selective impairment to the reading aloud of irregularly spelled words particularly if the words are low in frequency and have an abstract meaning e.g., indict. This impairment is accompanied by a preserved ability to read regularly spelled words and nonwords e.g. *zint*. Surface dyslexic reading is characterised by regularisation errors when reading word components e.g. yacht is pronounced 'ya/tch/ed'. Acquired surface dysgraphic patients misspell irregularly spelled words e.g. yacht → YOT and make homophone confusions in writing e.g. write the word *stake* when asked to write the sentence "He had steak for dinner". The opposite pattern of reading impairment is acquired phonological dyslexia, which refers to impaired reading of nonwords together with a preserved ability to read irregular and regular

ISSN 0953-4180/05/$17.00 © 2005 – IOS Press and the authors. All rights reserved words. Phonological dysgraphia refers to poor spelling of nonwords accompanied by preserved spelling of irregular and regular words. Acquired deep dyslexia is similar to phonological dyslexia except that patients produce errors that are semantic e.g. arm read as "finger", visual e.g. bus read as "brush" and morphological e.g. run read as "running". Deep dysgraphic patients make analogous errors in spelling e.g., "symphony" → orchestra. These patterns of acquired dyslexia and dysgraphia have been reported in other languages including Arabic [5], Chinese [32–34,57,59–61], Dutch [16], Finnish [31], French [5,6,23,36,55], Japanese [22,46, 47] Italian [2,35,40,41] and Spanish [3,10,11,13–15, 19,28–30,51]. Although the characteristics of reading and writing disorders vary across scripts, these reports reveal dissociable symptoms of acquired dyslexia and dysgraphia in quite different languages.

Coltheart et al. [9] proposed a 'multi-route' model to explain oral reading and acquired disorders of reading in English. The model assumes a *lexical semantic* pathway available for reading aloud known words and a *direct lexical* pathway that can read words without contacting the meaning of the word. Coltheart et al. [9] also assume a third *nonlexical grapheme to phoneme* route that is mandatory for reading nonwords such as *zint* and is available for reading regular words but cannot read irregular words correctly.

Plaut et al. [49] proposed a different model based on the connectionist principles of sub-symbolic processing. Their model assumes bi-directional pathways for reading and for spelling (see [53]). However, connectionist models eschew whole word representations in favour of subword components in onset, vowel and coda positions. Plaut et al. [49] use the terms *semantic* and *phonological* pathways instead of lexical-semantic and nonlexical grapheme to phoneme pathways respectively. The key distinction between the dual-route and connectionist models is that the former assume independent direct lexical and semantic pathways whereas connectionist models do not. Plaut et al. [49] also assume that reading (and spelling) of nonwords proceeds via the phonological pathway by a process of analogy with existing subword representations.

The models give contrasting accounts of acquired surface, phonological and deep dyslexia in English and can in principle explain these phenomena in other scripts also [5]. According to Coltheart [9] surface dyslexia results from damage to the direct lexical and/or lexical semantic pathways leading to over-reliance on the nonlexical route for reading aloud. This explains the tendency towards regularisation of irregular words using knowledge of grapheme-phoneme representations. Phonological dyslexia results from impairment to the nonlexical pathway together with preserved direct lexical and lexical semantic pathways. This explains the selective inability to read nonwords. Deep dyslexia might arise from the loss of the lexical and nonlexical pathways so reading is exclusively semantic. Surface dysgraphia could result from loss of the lexical-semantic pathway and phonological/deep dysgraphia from a loss of the nonlexical pathway [27]. According to Plaut et al. [49] surface dyslexia results from impairment to the semantic pathway due to damage to representations in semantic memory or to the bidirectional mappings linking word knowledge and phonological representations. Similarly, surface dysgraphia could arise from damage to semantic memory or mappings between semantics and orthography [24,25,27]. This leads to an over-reliance on the phonological pathway for reading and spelling. Phonological dyslexia and dysgraphia result from damage to the phonological pathway, which can be abolished in deep dyslexia [20]. Hillis and Caramazza [26] also developed a summation account of reading (and spelling) that assumes a basic dual route architecture, which is described in detail in papers in this issue (e.g. Shu and colleagues).

Although the models make different predictions about the cause of impairment in acquired dyslexia and dysgraphia, each model assumes there can be selective damage to functionally independent pathways linking orthography and phonology. Thus all models can explain the variety of reading and writing disorders in English speaking patients. The models have also been applied to explaining disorders of reading and writing observed in other languages and bilingual speakers – even in languages with distinctive orthographies. This point is illustrated by the report of an English/Cantonese biscriptal patient by Eng and Obler [17] who produced similar errors in English and Chinese scripts and also by the report of a French/Arabic case by Beland and Mimouni [5] who was deep dyslexic when reading in both the Latin (French) and Semitic (Arabic) alphabets (see also [18,50]).

The aim of this Special Issue is to describe communication disorders in patients who speak Cantonese, French, German, Hebrew, Mandarin, Spanish and Turkish. These languages were selected because they differ on critical linguistic dimensions (grammar, orthography, phonology) and although each dimension is important for understanding the disorders of communication in each language, the focus of this issue is selective disorders of reading and writing given cognitive neuropsychological models are mostly concerned with how the relationship between orthography, phonology and meaning impacts on acquired dyslexia and dysgraphia. The sample of languages discussed here also offers the opportunity of new insights into how different language environments – in terms of the script that has evolved in the environment – can have impact on brain function. This is an important question in cognitive neuroscience.

An initial step to understanding acquired dyslexia and dysgraphia across scripts is to report the phenomena observed in the neurology clinic. However even if a disorder can be interpreted with existing cognitive models, the aim of reporting cases in different languages should not be to support a "universal" model of reading and writing but instead serve as a basis for hypothesising about disorders *within* a language. Coltheart and Perry [8] made this point by arguing that to assume similarity in disorders of reading across scripts is a "kind of scientific cultural imperialism that runs the risk of obscuring important differences between scripts" p. 55. This may preclude insights that can teach us about the unique characteristics of reading (and spelling) across languages. Although this view is not universally held [5,17,22,28–36,40,44–48,50,57, 59–61], it is vital to interpret acquired disorders of reading and writing by considering differences between languages.

Linguists have long recognized that languages cannot be characterized into simple categories. However, one useful distinction for the present purposes is between alphabetic and non-alphabetic scripts. Alphabetic scripts use a finite number of symbols that can be combined to produce an infinite number of words. French, German, Spanish and Turkish all use printed letters or letter clusters to convey the pronunciation of words. Some alphabetic scripts are transparent because orthography conveys the pronunciation of a word in an unambiguous way. Turkish is the prime example of a transparent script because each letter is read in the same way and sounds are spelled in the same way in all words. Spanish is also a transparent script but is less transparent than Turkish. This is because Spanish contains some ambiguous pronunciations of letter combinations and some sounds cannot be spelled correctly without knowledge of the word context (e.g. b and v). German and French are less transparent than Spanish and Turkish, but French is the most opaque of these as the script contains vowel combinations with ambiguous pronunciations and spelling of vowel combinations requires knowledge of word context. Of course English is the least transparent of all European alphabetic scripts as irregular words e.g. *yacht, pint* and *aisle* require lexical knowledge for reading and spelling [56].

Hebrew uses an alphabetic script that is quite transparent for beginning readers. Children are taught to read using twenty-two symbols and five vowel sounds that are marked with diacritic symbols in print. However, skilled readers of Hebrew must learn to read written symbols as a series of consonants with no diacritic markers (called unpointed script) as these are omitted in most texts (books, newspapers and magazines). This is akin to having to decipher whether the printed letter sequence *fr* refers to for, far, four fir fur or fore, thus making reading in Hebrew rather like a guessing game, requiring considerable contextual knowledge [58]. Hebrew script is therefore more opaque than other alphabetic scripts for skilled readers because the mappings between orthography and phonology require morphological knowledge of the language. Hebrew and Arabic have been labeled as deep orthographies because the orthographic features require semantic processing [21].

In contrast to Indo-European languages, Japanese and Chinese (which include Cantonese and Mandarin/Putonghua) use non-alphabetic scripts. Non-alphabetic scripts are usually defined as logographic because the basic unit of writing is associated with a unit of meaning (the morpheme) in the spoken language. Each of these languages uses a large number of symbols called characters (there are over 50,000 Chinese characters) that uniquely represent an individual word or small number of words. As printed letters are not available to convey the pronunciation of words, mappings between orthography and phonology are opaque (although there are some phonetic radicals that denote a common pronunciation across characters). Another feature of non-alphabetic scripts is that the mappings between orthography and meaning are transparent in many characters, whereas mappings between orthography and meaning in alphabetic scripts are relatively opaque.

The papers in this Special Issue are arranged around the theme of how selective disorders of reading and writing are manifest according to the transparency of the script. In the first group of papers the authors consider reading and writing in transparent scripts. Raman reports a patient with deep dysgraphia in Turkish which includes poor nonword spelling, effects of lexical variables e.g. imageability and semantic errors in written word production. As described above, deep dysgraphia in English is assumed to reflect loss of the nonlexical spelling pathway with over-reliance on spelling via the lexical-semantic pathway. This is plausible in opaque scripts given the need to retrieve lexical knowledge for the spelling of many words. It is more surprising to find an effect of lexical variables on spelling in a completely transparent writing system. Raman's case illustrates the value of data in behavioural neurology as individual cases can reveal phenomena that would not be obvious when investigating normal spelling in Turkish, which is assumed to be effortless. Ferreres and colleagues pursue this theme with a counterintuitive finding from Spanish. As Spanish contains few irregularities in orthography, it seems at first redundant to examine impairments in the lexical semantic reading system. However, by testing unique features of orthographic knowledge and also reading speed in Spanish, the authors make a persuasive argument for why lexical reading is necessary for Spanish speakers. They report patient MM, who manifests a pattern of impaired reading that is similar to the symptoms of surface dyslexia in English. For example the use of a written accent for marking syllable stress in Spanish normally allows the reader to decode printed words for meaning. MM has lost the ability to access word meanings using these orthographic cues and as a consequence his comprehension is impaired. This finding suggests that despite the transparent orthography, normal oral reading in Spanish requires retrieval of lexical knowledge. In the paper by Davies and Cuetos this view is strengthened by their report of a Spanish speaker who shows the converse pattern i.e. deep dyslexia. As described above deep dyslexia in other languages is assumed to result from loss of the non-lexical reading pathway and over-reliance on a lexical semantic pathway and so deep dyslexia in Spanish reveals the operation of lexical-semantic reading. All the patients reported in this group show that the pattern of double dissociations found between lexical semantic pathways and nonlexical pathways that are assumed to be available for oral reading and spelling in English can also be observed in languages that have transparent orthographies.

The next set of papers report patients with acquired dyslexia in German, acquired dysgraphia in French and acquired and development dyslexia in Hebrew. Each paper describes how acquired dyslexia or dysgraphia could be manifest in those languages. Stenneken and colleagues report a patient US who is unable to retrieve phonological output from printed words in German – a relatively transparent orthography – but who can access the meaning of these words 'directly'. They argue that phonological mediation may not be mandatory for word recognition in transparent scripts as assumed by many in the literature. De Partz and colleagues report a French speaker who produces errors in writing that are similar to the errors observed in patients who have acquired dysgraphia in English. They provide evidence for two distinct types of letter representation in French: symbolic letter representation at the graphemic level and representations of component graphic strokes at the letter form level. Friedmann and Gvion report a pattern of neglect dyslexia for left hand letters in Hebrew. One distinctive property of Hebrew is that it is read from right to left rather than left to right as in other alphabetic scripts. This means that left word based neglect dyslexia causes impairment to final rather than first letters.

In the final group of papers the authors consider acquired dyslexia and dysgraphia in languages with non-alphabetic scripts. Chinese scripts are the focus since we know so little about these widely spoken languages. Cantonese speakers use traditional characters in Hong Kong whereas Putonghua speakers in Mainland China use a simplified script (to complicate matters, Taiwanese individuals who speak Putonghua learn to read traditional Mandarin characters). It is important to realize that Putonghua and Cantonese are quite different languages for reading. This is because not only is the syntax of each language different but the meaning of some characters differs in Cantonese and Mandarin as well.

Cognitive neuropsychological studies of Chinese speaking aphasic patients have become more common in the last decade [32–34]. Yin reviews the reports of patients in the literature with a focus on the characteristics of surface dyslexia in Chinese. It is of some interest to compare the pattern of reading comprehension errors observed in surface dyslexia in Chinese to the homophone errors reported in Spanish speakers (MM reported by Ferreres this volume). In both scripts the comprehension of phonologically ambiguous words (homophones) – which require orthographic knowledge to be understood (pear-pair) – is impaired in surface dyslexia. In both Chinese and Spanish this is assumed to reflect damage to a lexical-semantic reading pathway. A comparison of surface dyslexic symptoms across scripts therefore shows that the same phenomenon can be observed in alphabetic and non-alphabetic scripts. This suggests a common mechanism might be the cause of impaired reading comprehension in both languages. Crucially, this insight was achieved by highlighting characteristics of transparent and opaque orthographies, which despite differences, appear to involve common mechanisms for reading comprehension. The penultimate paper by Law and colleagues describes a Cantonese speaker who is unable to understand the meaning of characters but correctly reads the same characters aloud similar to patients reported in English and Japanese. They also argue that naming errors produced by patient YKM reveals the structure of the phonological output lexicon in Chinese. The last paper by Shu and colleagues reports a Putonghua speaker SJ with acquired dyslexia who can write the characters that she cannot read. The data from SJ suggests that phonological mediation may not be necessary for written word production in Chinese. The data from both studies shows reading and writing systems are dissociable in Chinese.

Several papers in the Special Issue converge on the same conclusion. Studies of single cases of acquired dyslexia and dysgraphia suggest at least two pathways are used for normal reading and writing across different scripts. If the pathways are labeled lexical semantic and nonsemantic then the differences between scripts – such as whether reading via nonlexical mechanisms is necessary – informs the development of cognitive models of reading and writing. The issue of whether additional mechanisms are needed to explain reading and writing for a given language is then a hypothesis that can be examined within that language. For example, it is quite appropriate to ask whether a nonlexical pathway is necessary to read nonwords in alphabetic scripts [9,13] but this question is redundant for non-alphabetic scripts given that reading is always a lexical event [61]. Similarly, it is appropriate to ask if a direct lexical pathway is used to read irregular words in English and characters in Chinese, but this is not an appropriate question to ask about reading in a transparent script such as Turkish, as there are no irregular words [50]. According to this view of reading and writing across scripts it is reasonable to investigate acquired dyslexia and dygraphia in other languages in order to test hypotheses derived from a dual route framework. However, the unique properties of a script will require some modification to this basic framework. In closing, I would like to express my gratitude to colleagues who provided expertise in the reviewing and the editing of papers for this Special Issue: Judit Druks, Liory Fern-Polk, Silvia Genari, Mira Goral, Argye Hillis, Connie Ho, Carolina Iribarren, Baris Kabak, Sam Po Law, Qian Luo, Steve Majerus, Loraine Obler, Benjamin Parris, Conrad Perry, Marie-Josephe Tainturier, Carolyn Wilshire, Max Wilson and Jo Ziegler. Thanks also to the contributors for their insights into acquired disorders of reading and writing across scripts.

### References

[1] M. Albert and L.K. Obler, *The Bilingual Brain: Neuropsychological and Neurolinguistic Aspects of Bilingualism,* Academic Press, New York, 1978.

[2] P. Angelelli, A. Judica, D. Spinelli, P. Zoccolotti and C. Luzzatti, Characteristics of writing disorders in Italian dyslexic children, *Cognitive and Behavioral Neurology* **17**(1) (2004), 18–31.

[3] A. Ardilla, Errors resembling semantic paralexias in Spanish speaking aphasics, *Brain and Language* **41** (1991), 437–445.

[4] M. Behrmann and B. Bub, Surface dyslexia and dysgraphia: dual routes, single lexicon, *Cognitive Neuropsychology* **9** (1992), 209–251.

[5] R. Beland and Z. Mimouni, Deep dyslexia n the two languages of an Arabic/French bilingual patient, *Cognition* **82** (2001), 77–126.

[6] D. Cardebat, M. Puel, J.F. Demonet and J.L. Nespoulous, Les differentes "boites" de la repetition: Analyse d'un case de dysphasie profonde, *Revue de Neuropsycholgie* **1** (1991), 215–232.

[7] M. Coltheart, Writing systems and reading disorders, in: *Orthographies and Reading: Perspectives from Cognitive Psychology, Neuropsychology, and Linguistics,* L. Henderson, ed., Erlbaum, London, 1984.

[8] M. Coltheart and C. Perry, Reading Chinese: Dual route theory and acquired dyslexia, in: *Advancers in Theoretical Issues and Cognitive Neuroscience Research of the Chinese Language,* H. Kao and C. Lin, eds, The University of Hong Kong, Hong Kong, 1998.

[9] M. Coltheart, K. Rastle, C. Perry, R. Langdon and J. Ziegler, DRC: A dual route cascaded model of visual word recognition and reading aloud, *Psychological Review* **108**(1) (2001), 204–256.

[10] F. Cuetos, Writing processes in a shallow orthography, *Reading and Writing: An Interdisciplinary Journal* **5** (1993), 17–28.

[11] F. Cuetos and E. Labos, The autonomy of the orthographic pathway in a shallow language: Data from an aphasic patient, *Aphasiology* **15**(4) (2001), 333–342.

[12] F. Cuetos and A. Ellis, Visual paralexias in a Spanish-speaking patient with acquired dyslexia: A consequence of visual and semantic impairments? *Cortex* **35**(5) (1999), 661–674.

[13] F. Cuetos, F. Valle-Arroyo and M. Suarez, A case of phonological dyslexia in Spanish, *Cognitive Neuropsychology* **13**(1) (1996), 1–24.

[14] F. Cuetos, T. Martinez, C. Martinez, C. Izura and A. Ellis, Lexical processing in Spanish patients with probable Alzheimer's disease, *Cognitive Brain Research* **17**(3) (2003), 549–561.

[15] S. Defior, F. Justica and F. Martos, The influence of lexical and sublexical variables in normal and poor Spanish readers, *Reading and Writing* **8** (1996), 487–497.

[16] H.F.A. Diesfeldt, Impaired and preserved semantic memory functions in dementia, in: *Memory functioning in dementia,* L. Blackman, ed., Elsevier Science Publishers, Amsterdam, 1992.

[17] N. Eng and L.K. Obler, Acquired dyslexia in a biscript reader following traumatic brain injury: A second case, *Topics in Language Disorders* **22**(5) (2002), 5–19.

[18] F. Fabbro, *The neurolinguistics of bilingualism: An Introduction,* Psychology Press, Psychology Press, Hove, 1999.

[19] A.R. Ferrreres and G. Miravalles, The production of semantic paralexias in a Spanish speaking aphasic, *Brain and Language* **49** (1995), 153–172.

[20] R.B. Friedman, Recovery from deep alexia to phonological alexi: Points on a continuum, *Brain and Language* **52** (1996), 114–128.
[21] R. Frost, Toward a strong phonological theory of visual word recognition: True issues and false trails, *Psychological Bulletin* **123** (1998), 71–99.
[22] T. Fushimi, K. Komori, M. Ikeda, K. Patterson, M. Ijuin and H. Tanabe, Surface dyslexia in a Japanese patient with semantic dementia: evidence for similarity-based orthography-to-phonology translation, *Neuropsychologia* **41**(12) (2003), 1644–1658.
[23] J. Goldblum, Word comprehension in surface dyslexia, in: *Surface dyslexia*, K. Patterson, J. Marshall and M. Coltheart, eds, Lawrence Erlbaum Associates, London, 1985.
[24] N.L. Graham, K. Patterson and J.R. Hodges, Progressive dysgraphia: Co-occurrence of central and peripheral impairments, *Cognitive Neuropsychology* **14** (1997), 975–1005.
[25] N.L. Graham, K. Patterson and J.R. Hodges, The impact of semantic memory impairment on spelling: evidence from semantic dementia, *Neuropsychologia* **38** (2000), 143–163.
[26] A.E. Hillis and A. Caramazza, Converging evidence for the interaction of semantic and sublexical phonological information in accessing lexical representation for spoken output, *Cognitive Neuropsychology* **12** (1995), 187–227.
[27] G. Houghton and M. Zorzi, Normal and impaired spelling in a connectionist dual-route architecture, *Cognitive Neuropsychology* **20**(2) (2003), 115–162.
[28] I.C. Iribarren, G. Jarema and A.R. Lecours, The assessment of surface dyslexia in a regular orthography, Spanish: A case study, *Brain and Cognition* **32**(2) (1996), 196–198.
[29] I.C. Iribarren, G. Jarema and A.R. Lecours, Lexical reading in Spanish: Two cases of phonological dyslexia, *Applied Psycholinguistics* **20**(3) (1999), 407–428.
[30] I.C. Iribarren, G. Jarema and A.R. Lecours, Two different dysgraphic syndromes in a regular orthography, Spanish, *Brain and Language* **77**(2) (2001), 166–175.
[31] M. Laine, P. Neimi, J. Neimi and P. Koivuselkä-Sallinen, Semantic errors in deep dyslexia, *Brain and Language* **38** (1990), 207–214.
[32] S.P. Law and B. Or, Nonsemantic pathways of reading and writing Chinese: Data from a Cantonese-speaking brain-damaged patient, *Brain and Language* **74**(3) (2000), 524–526.
[33] S.P. Law and B. Or, A case study of acquired dyslexia and dysgraphia in Cantonese: evidence for nonsemantic pathways for reading and writing Chinese, *Cognitive Neuropsychology* **18**(8) (2001), 729–748.
[34] S.P. Law, Impairment to phonological processes in a Cantonese-speaking brain-damaged patient, *Aphasiology* **18**(4) (2004), 373–388.
[35] S. Luzzi, M. Bartolini, M. Coccia, L. Provinciali, M. Piccirilli and J.S. Snowden, Surface dysgraphia in a regular orthography: Apostrophe use by an Italian writer, *Neurocase* **9**(4) (2003), 285–296.
[36] S. Majerus, F. Leku, M. Van de Linden and E. Salmon, Deep dysphasia: Further evidence on the relationship between phonological short term memory and language processing impairments, *Cognitive Neuropsychology* **18** (2001), 385–410.
[37] J.C. Marshall and F. Newcombe, Syntactic and semantic errors in paralexia, *Neuropsychologia* **4** (1966), 169–176.
[38] J.C. Marshall and F. Newcombe, Patterns of paralexia: a psycholinguistic approach, *Journal of Psycholinguistic Research* **2** (1973), 175–200.
[39] L. Menn and L.K. Obler, Cross-linguistic data and theories of agrammatism, in: *Agrammatic aphasia: A cross-Language Narrative Sourcebook*, L. Menn and L.K. Obler, eds, Amsterdam: John Benjamins Publishing Company, 1990, pp. 1369–1389.
[40] M. Miozzo and P. de Bastiani, The organization of letter-form representations in written spelling: Evidence from acquired dysgraphia, *Brain and Language* **80**(3) (2002), 366–392.
[41] G. Miceli and A. Caramazza, The assignment of word stress in oral reading: Evidence from a case of acquired dyslexia, *Cognitive Neuropsychology* **10**(3) (1993), 273–296.
[42] L.K. Obler and K. Gjerlow, *Language and the Brain*, Cambridge University Press, 2000.
[43] M. Paradis, Multilingualism and aphasia in G Blanken, in: *Linguistic Disorders and Pathologies*, J. Dittman, H. Grimm, J. Marshall and C.W. Wallesch, eds, Berlin: Walter de Gruyterm, 1993.
[44] K. Patterson, Basic processes of reading: Do they differ in Japanese and English? *Japanese Journal of Neuropsychology* **6** (1990), 4–14.
[45] K. Patterson and J.R. Hodges, Deterioration of word meaning: Implications for reading, *Neuropsychologia* **30** (1992), 1025–1040.
[46] K. Patterson, T. Suzuki, T. Wydell and S. Sasanuma, Progressive aphasia and surface alexia in Japanese, *Neurocase* **1** (1995), 155–165.
[47] K. Patterson, S. Okada and T. Suzuki, Fragmented words: A case of late-stage progressive aphasia, *Neurocase* **4**(3) (1998), 219–230.
[48] K. Patterson and M.A. Lambon Ralph, Selective disorders of reading? *Current Opinion in Neurobiology* **9** (1999), 235–239.
[49] D.C. Plaut, J.D. McClelland, M.S. Seidenberg and K. Patterson, Understanding normal and impaired word reading: Computational principles in quasi-regular domains, *Psychological Review* **103** (1996), 56–115.
[50] I. Raman and B.S. Weekes, Acquired dyslexia in a Turkish-English speaker, *Annals of Dyslexia* **55**(1) (2005), 71–96.
[51] A. Ruiz, A.I. Ansaldo and A.R. Lecours, Two cases of deep dyslexia in unilingual Hispanophone aphasics, *Brain and Language* **46**(2) (1994), 245–256.
[52] S. Sasanuma, N. Sakuma and K. Kitano, Reading Kanji without semantics: evidence from a longitudinal study of dementia, *Cognitive Neuropsychology* **9**(6) 1992, 465–486.
[53] M.S. Seidenberg and J.L. McClelland, A distributed, developmental model of word recognition and naming, *Psychological Review* **96** (1989), 523–568.
[54] W.T. Siok, C.A. Perfetti, Z. Jin and L.H. Tan, Biological abnormality of impaired reading is constrained by culture, *Nature* **431** (2004), 71–76.
[55] S. Valdois, S. Cabonnel, D. David, S. Rouset and J. Pellat, Confrontation of PDP models and dual route models through the analysis of a case of deep dysphasia, *Cognitive Neuropsychology* **12** (1995), 681–724.
[56] B. Weekes and M. Coltheart, Surface dyslexia and surface dysgraphia: Treatment studies and their theoretical implications, *Cognitive Neuropsychology* **13** (1996), 277–315.
[57] B. Weekes and H.Q. Chen, Surface dyslexia in Chinese, *Neurocase* **5**(2) (1999), 161–172.
[58] B. Weingrod, Language and treatment in Israel, in: *The International Book of Dyslexia*, R. Salter and I. Smythe, eds, WDNF, London, 1997.
[59] W.G. Yin, *On reading Chinese characters*, Unpublished PhD thesis, University College London, 1991.
[60] W.G. Yin and B. Butterworth, Deep and surface dyslexia in Chinese, in: *Language Processing in Chinese*, H.C. Chen and

O.J.L. Tzeng, eds, Elsevier Science Publishers, Amsterdam, 1992, pp. 349–366.
[61] W.G. Yin and B.S. Weekes, Dyslexia in Chinese: Clues from cognitive neuropsychology, *Annals of Dyslexia* **53** (2003), 255–279.
